# Evaluation of CT and MRI Radiomics for an Early Assessment of Diffuse Axonal Injury in Patients with Traumatic Brain Injury Compared to Conventional Radiological Diagnosis

**DOI:** 10.1007/s00062-025-01507-6

**Published:** 2025-03-07

**Authors:** Anna-Katharina Meißner, Robin Gutsche, Lenhard Pennig, Christian Nelles, Enrico Budzejko, Christina Hamisch, Martin Kocher, Marc Schlamann, Roland Goldbrunner, Stefan Grau, Philipp Lohmann

**Affiliations:** 1 Dept. of General Neurosurgery, Center for Neurosurgery, Faculty of Medicine and University Hospital Cologne, University of Cologne, Kerpener St. 62, 50935 Cologne, Germany; 2 Inst. of Neuroscience and Medicine (INM-4), Research Center Juelich (FZJ), Juelich, Germany; 3 Inst. for Diagnostic and Interventional Radiology, Faculty of Medicine and University Hospital Cologne, University of Cologne, Cologne, Germany; 4 Dept. of Neurosurgery, Klinikum Fulda, Academic Hospital of the University of Marburg, Fulda, Germany; 5 Dept. of Stereotactic and Functional Neurosurgery, Center for Neurosurgery, Faculty of Medicine and University Hospital Cologne, University of Cologne, Cologne, Germany; 6 Dept. of Nuclear Medicine, University Hospital RWTH Aachen University, Aachen, Germany

**Keywords:** Machine learning, Artificial intelligence (AI), Neuroimaging, Predilection areas, AI-based decision support

## Abstract

**Background:**

De- and acceleration traumata can cause diffuse axonal injury (DAI) in patients with traumatic brain injury (TBI). The diagnosis of DAI on CT is challenging due to the lack of structural abnormalities. Radiomics, a method from the field of artificial intelligence (AI) offers the opportunity to extract additional information from imaging data. The purpose of this work was the evaluation of the feasibility of radiomics for an improved diagnosis of DAI in comparison to conventional radiological image assessment.

**Methods:**

CT and MR imaging was performed in 42 patients suspicious of DAI due to the clinical state, and two control groups (*n* = 44;42). DAI was diagnosed by experienced neuroradiologists. Radiomics features were extracted using a standardized MRI-based atlas of the predilection areas for DAI. Different MRI and CT based models were trained and validated by five-fold cross validation. Diagnostic performance was compared to the reading of two experienced radiologists and further validated in an external test dataset.

**Results:**

The MRI and CT models showed significant differences in radiomics features between patients with DAI and controls. The developed MRI based random forest classifier yielded an accuracy of 80–90%. The best performing CT model yielded an accuracy of 88% in the training data and 70% in the external test data. The results were comparable to conventional image analysis which achieved an accuracy of 70–81% for CT-based diagnosis.

**Conclusion:**

MRI- and CT-based radiomics analysis is feasible for the assessment of DAI. The radiomics classifier achieved equivalent performance rates as visual radiological image diagnosis. Especially a radiomics based CT classifier can be of clinical value as a screening and AI-based decision support tool for patients with TBI.

## Introduction

**Fig. 1:**
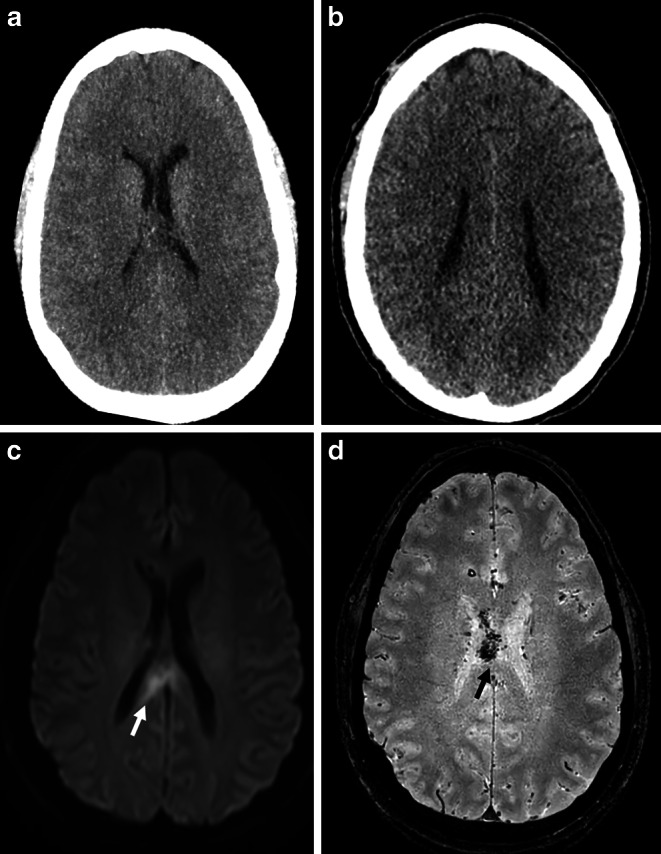
CT scan (**a** + **b**) and MRI (**c** DWI, **d** SWI) of a patient with DAI at different levels in axial view. As the CT lacks distinct structural abnormalities, MRI sequences show a higher sensitivity with typical micro bleedings (black arrow) and diffusion impairment (white arrow) in the predilection areas for DAI. *DAI* Diffuse axonal injury, *DWI* Diffusion-weighted MRI, *SWI* Susceptibility-weighted MRI

Traumatic brain injury (TBI) is one of the main causes of death and morbidity, especially in young adults, but also in the growing elderly population. Worldwide more than 50 million TBIs per year are reported, illustrating that TBI remains one of today’s main medical and socioeconomical challenges [1–3]. De- and acceleration traumata can cause shearing of the white matter tracts and result in diffuse axonal injury (DAI) in up to 40–75% of patients with mild to severe TBI [4–6]. These axonal injuries typically occur in certain predilection areas as the brain stem, thalamus, corpus callosum and corona radiata and seem to be one of the most common and important features in TBI [4, 5, 7]. Patients with DAI often present with immediate onset coma and tend to have an increased risk for long-term morbidity, neuropsychological deficits, and the development of neurodegenerative diseases [7–9]. The standard diagnostic procedure in the acute phase of TBI is a cranial computed tomography (CT) scan. Minor imaging findings as micro bleedings and diffuse brain edema can be rated as indirect signs for DAI. But due to the lack of distinct structural abnormalities, the diagnosis of DAI on CT is challenging [10–12] (see Fig. 1). To overcome this limitation, in cases with clinically suspected DAI, MR imaging is necessary. Diffusion (DWI)—and susceptibility-weighted (SWI)—sequences seem to be particularly valuable to detect white matter injuries [10, 13]. Nevertheless, MRI is time consuming and cost expensive and not always suitable for instable ICU patients. Therefore, DAI remains underdiagnosed and the development of sensitive methods for an early and reliable CT-based diagnosis are of high clinical value.

Radiomics is a method from the field of artificial intelligence aiming at the extraction of quantitative features from routinely acquired imaging data, not accessible by conventional image analysis [14, 15]. Especially in combination with clinical parameters, radiomics can be used to develop mathematical models to support clinical decision-making [16–18]. Up to now, in the field of neurology and neurosurgery, radiomics is mainly used addressing neurooncological questions. Applications for TBI and especially DAI are rare.

In a study from Hollie et al., MRI based texture analysis, as a type of basic radiomics, was feasible to detect significant structural differences between the hemispheres in patients with mild TBI in comparison to healthy controls [19, 20]. With radiomics analysis of diffusion-kurtosis imaging (DKI), statistically significant feature differences were observed between patients with DAI and healthy controls [21]. These previous studies extracted limited features from small regions of interest and further validation of the results in comparison to conventional image analysis and in an independent test dataset is lacking. Up to now, there is no data available on CT-based radiomics analysis in patients with DAI.

Here, we evaluated the potential of MRI radiomics as a proof-of-concept study for the diagnosis of DAI in patients with severe TBI. We then transferred the concept to consecutively evaluate the potential of CT radiomics for this task. The validity and performance of the developed models were evaluated in comparison to experienced radiologists and the CT model was furthermore tested in an independent data set from a second institution.

## Patients and Methods

### Ethics Statement

The present study was conducted according to the guidelines of the Declaration of Helsinki, and the retrospective analysis of data was approved by the Ethics Committees of the University Hospital Cologne, Germany (approval number 21-1344).

### Patients and Controls

From 2011 to 2023, we retrospectively identified patients with clinically suspected DAI which was confirmed on MRI from the Neurosurgical Centers of the University Hospital Cologne and Hospital Fulda, Germany. MR imaging reports were used as ground truth. Patients received a CT scan on the point of admission and follow-up MRI including DWI after trauma. Exclusion criteria were according to the Marshall score [22] (i) large intraparenchymal hematoma > 25 cm^3^ (ii) major traumatic subarachnoid bleeding and (iii) midline shift > 5 mm in the CT scan at admission. Clinical data were retrieved from an electronic database and patients’ paper charts. We recorded gender, age, GCS (severity of TBI), loss of consciousness and pupillary defect at the point of admission. Two control groups (one for CT and one for MRI) without previous history of TBI and regular imaging including the required MRI sequences were retrospectively identified from the hospital imaging data bases.

### MR Imaging

MR imaging procedures for diagnosis of DAI at both neurosurgical centers comprised T2-weighted sequences, fluid attenuated inversion recovery (FLAIR) imaging, DWI and SWI or T2* sequences. At the university hospital cologne, sequences for further analysis were performed at 3 T (Ingenia, Philips Healthcare, Best, The Netherlands) with following parameters: DWI: single-shot, spin echo planar imaging with 30 slices with a thickness of 5 mm and an increment of 0.5 mm (TR 3330 msec, TE 76 msec, flip angle 90°, acquisition matrix 168 × 111), obtaining baseline images (b = 0 sec/mm^2^) and images with enabled diffusion gradient along each of 3 orthogonal directions (b = 1000 sec/mm^2^). Diffusion trace maps were computed from the isotropic diffusion image and were used to estimate the apparent diffusion coefficient. The parameters for SWI were: 3D sequence with 263 slices with a thickness of 0.6 mm and an increment of 0.6 mm (TR 44.76 msec, TE 24.38 msec, flip angle 10°, acquisition matrix 224 × 223) and the parameters for FLAIR were: 3D sequence with 308 slices with a thickness of 1.12 mm and an increment of 1 mm (TR 4800 msec, TE 340 msec, TI 1650 msec, flip angle 90°, acquisition matrix 224 × 223).

At the hospital Fulda, sequences for further analysis were performed at 3 T (Ingenia, Philips Healthcare, Best, The Netherlands) with following parameters: DWI: single-shot, spin echo planar imaging with 35 slices with a thickness of 4 mm and an increment of 0.4 mm (TR 3946 msec, TE 71 msec, flip angle 90°, acquisition matrix 152 × 122), obtaining baseline images (b = 0 sec/mm2) and images with enabled diffusion gradient along each of 3 orthogonal directions (b = 1000 sec/mm2). Diffusion trace maps were computed from the isotropic diffusion image and were used to estimate the apparent diffusion coefficient. The parameters for venous BOLD were: 3D sequence with 250 slices with an increment of −0.5 mm (TR 21 msec, TE 27 msec, flip angle 10°, acquisition matrix 220 × 181) and the parameters of the FLAIR were: 3D sequence with 350 slices with a thickness of 1.12 mm and an increment of 0 mm (TR 4880 msec, TE 280 msec, TI 1650 msec, angle 40°, acquisition matrix 224 × 224).

### CT Imaging

CT images at the University Hospital Cologne were acquired using a 256 slice helical CT scanner (Brilliance iCT 256, Philips Healthcare, Best, The Netherlands) with following parameters: tube voltage 120 kVp, tube current-time product 320 mAs, pitch factor 0.4, rotation time 0.4 sec, collimation 40 × 0.625 mm, slice thickness 1 mm, increment 0.5 mm, matrix 512 × 512 and CTDI_vol_ 44.2 mGy. Images were reconstructed using an iterative reconstruction algorithm (iDose, kernel UB, Philips Healthcare).

CT images at the Hospital Fulda were acquired using a 128 slice helical CT scanner (Siemens Somatom Definition EDGE 128) with following parameters: tube voltage 120 kVp, tube current-time product 209–330 mAs, pitch factor 0.55, rotation time 1 sec, collimation 128 × 0.66 mm, slice thickness 1 mm, increment 1 mm, matrix 256 × 256 and CTDi vol 29.65–55.12 mGy. Images were reconstructed using a maximum intensity projection.

### Standardized Brain Atlas for Predilection Areas of DAI

**Fig. 2:**
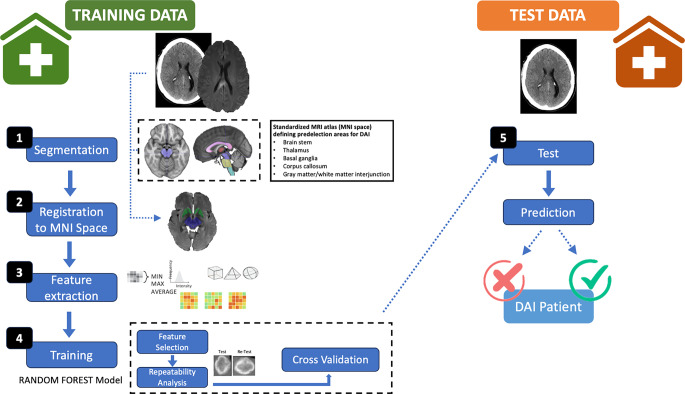
Workflow for development of the radiomics classifier

To define the target volume for radiomics feature extraction a standardized anatomical MRI atlas of the predilection areas for shearing injuries was developed using the open-source image analysis software ITK SNAP version 3.6 (http://www.itksnap.org/) [23]. Anatomical structures were manually outlined on a standardized MRI template (T1-weighted sequence, 1 mm slice thickness, MNI152 (Montreal Neurological Institute)) [24, 25]. The atlas comprised the following regions of interest (ROIs): brain stem divided in mesencephalon, pons and medulla oblongata, thalamus, basal ganglia, corpus callosum divided in rostrum, genus, corpus and splenium, circular regions of interest (1 cm diameter) at white/matter gray matter junctions in the frontal, temporal, parietal and occipital lobe (see Fig. 2).

### Image Preprocessing

Image preprocessing for MRI (DWI sequences) and CT was performed using the FSL toolbox version 5.0 (FMRIB Software Library, http://www.fmrib.ox.ac.uk/fsl) [25] the MIC-DKFZ HD-BET brain extraction tool (https://github.com/MIC-DKFZ/HD-BET) [16], the Advanced Normalization Tools ANTS version 2.1 (http://stnava.github.io/ANTs) [26], following the conversion of all images to NifTI format (dcm2niix, https://github.com/rordenlab/dcm2niix) [28]. After brain extraction using HD-BET, a nonparametric normalization algorithm for the removal of low frequency intensity nonuniformities (bias field) was performed using N4ITK [29]. MR image intensities were Z‑score normalized according to current recommendations [30]. All images were resampled to an isotropic voxel size of 1 × 1 × 1 mm^3^ and warped to the MNI space using the software PMOD (version 4.2) (PMOD Technologies Ltd Zurich, Switzerland; www.pmod.com). Afterwards, the atlas was applied for the definition of ROIs for further analysis.

### Radiomics Feature Extraction

Three basic group of mathematically predefined radiomics features were extracted from the ROIs in the DWI and CT scans of patients and controls using the open source package PyRadiomics (version 3.0.1) in Python [31], including 26 shape, 18 histogram and 75 textural features. Features were extracted on the original images as well as filtered versions of the images after wavelet transformation and Laplacian-of-Gaussian filtering, resulting in a total number of 1328 features per scan. Shape features such as volume and maximum diameter represent geometric relations in a ROI [16]. Histogram features such as entropy and kurtosis describe the distribution of individual pixel or voxel intensity values within the segmented ROI, without considering their spatial orientation and relationship [16, 17]. In contrast, textural features represent statistical relationships between intensity levels of neighboring pixels or voxels or groups of pixels or voxels, which are computed from descriptive matrices that already encode specific spatial relations between pixels or voxels in the original images such as the gray-level co-occurrence matrix (GLCM) [16, 26].

### Feature Repeatability

As described previously [27, 28], a test-retest analysis was performed to avoid the use of non-robust radiomics features. For this purpose, augmented versions of the original images were generated using the image perturbation method chain translation, noise, and volume adoption [29, 30]. Repeatability between radiomics features from the original and augmented images were evaluated by the intraclass correlation coefficient (ICC). Features were considered repeatable if the lower and upper limits of the ICC 95% confidence interval were in the range of 0.91 to 1.00 and selected for further analysis.

### Feature Selection and Standardization

After performing repeatability analysis, features were selected based on their importance. In random forest modeling, feature importances are calculated as the mean and standard deviation of the accumulation of impurity decrease within each tree (for ref, see [31]). We fitted the random forest model to the repeatable features of the training set from the University Hospital Cologne, prior dividing it into training and validation sets in a 70/30 ratio. Subsequently, the most important features were selected based on their significance for classification in the validation set.

Finally, radiomics features were standardized by subtracting the mean and dividing by the standard deviation. In MRI and CT based models, mean and standard deviation were based on the training set (University Hospital Cologne).

### Model Training and Testing

At first, for the proof-of-concept study, MRI based random forest models for the different ROIs were generated using the data set and DWI sequences from the University Hospital Cologne. Five-fold stratified cross validation (training and validation set in a 70/30 split ratio) was performed to evaluate the performance of the selected most important features and model in MRI. No test dataset was used for the MRI model as the MRI study was designed as proof-of-concept study to facilitate further CT analysis.

The concept was subsequently transferred to CT imaging and independent random forest models were trained using the CT dataset from the University Hospital Cologne. The training performance was also evaluated by five-fold stratified cross validation. Finally, the CT model was retrained on the complete training data set and then one time applied to the external CT test data set (Hospital Fulda). Importantly, the final model testing was performed blinded for the diagnosis of DAI, and the diagnostic performance of the classifier was assessed fully independent from the researchers involved in model development. The radiomics workflow is presented in Fig. 2. All processing steps were implemented in Python (sklearn, version 0.24.1).

### Visual Image Analysis

All included MRI (all sequences) and CT scans from patients and controls were retrospectively diagnosed for DAI by two experienced board-certified radiologists with at least 6 years of experience using a DICOM viewing platform (IMPAX, AGFA Health Care) blinded for clinical parameters and previous radiology reports. CT and MRI images were reviewed independently. The raters classified the images using a dichotomous categorization (DAI yes or no).

### Statistical Analysis

Descriptive statistics are provided as mean and standard deviation or median and range. The diagnostic performance of each classifier was evaluated by receiver operating characteristics analysis. To avoid experimental bias, the statistical analysis of the test dataset was performed by a researcher not involved in the model generation. Significant differences between features were tested using the two-tailed Student’s t‑test, with previous confirmation of a normal distribution by the Shapiro-Wilk test. For neuroradiological assessment diagnostic accuracy was calculated. Interrater reliability was assessed using Cohens Kappa. *P*-values of 0.05 or less were considered statistically significant. Statistical analyses were implemented in Python (Pingouin, version 0.3.9) [32] and IBM SPSS Statistics, version 29.

## Results

### Clinical Characteristics of Patients and Controls

A total of 47 patients (training dataset MRI and CT: 42; test dataset CT: 5) and 91 controls (MRI control group: 44; CT control group: training dataset: 42, test dataset: 5) from two major neurosurgical centers were retrospectively included in the study. 72% of patients presented with severe TBI (GCS < 8) and loss of consciousness in 81% of cases. Clinically suspected DAI was confirmed in all patients by multimodal MRI assessment by experienced neuroradiologists.

For MRI analysis, 42 patients (mean age, 33 ± 15; age range, 14–68; 11 females, 31 males) and 44 controls (mean age, 33 ± 15; age range, 14–81; 24 females, 20 males) from the Department of General Neurosurgery of the University Hospital of Cologne, Germany were included as training dataset.

For CT analysis, 39 patients from two neurosurgical centers were available. The training dataset comprised of 34 patients (mean age, 33 ± 15; age range, 14–68; 9 females, 25 males) and 42 controls (mean age, 58 ± 18; age range, 22–80; 26 females, 16 males) from the University Hospital of Cologne, Germany (8 patients from the total dataset had to be excluded due to insufficient accuracy of matching to the MNI space). As test dataset 5 patients (mean age, 30 ± 17; age range, 16–52; 1 female, 4 males) and 5 controls (mean age, 54 ± 19; age range, 30–81; 2 females, 3 males) from the Hospital Fulda, Germany were included. The mean time interval between the CT scan and MRI in the dataset from University Hospital Cologne was 23 ± 59 days (range, 0–317). Patient and control characteristics are summarized in Table 1.

**Table 1:** Patient and control characteristics

	Training Set—Cologne				Test Set—Fulda	
	Patients		Controls		Patients	Controls
**Imaging**	MRI (*n* = 42)	CT (*n* = 34)	MRI (*n* = 44)	CT (*n* = 42)	MRI + CT (*n* = 5)	CT (*n* = 5)
**Sex **(female/male)	11/31	9/25	24/20	26/16	1/4	2/3
**Age in years** (mean ± SD (range))	33 ± 15 (14–68)	33 ± 15 (14–68)	33 ± 15 (14–81)	58 ± 18 (22–80)	30 ± 17 (16–59)	54 ± 19 (30–81)
**Severity of TBI**						
Grade 1 (GCS 13–15)	7	6	–	–	1	–
Grade 2 (GCS 9–12)	3	2	–	–	0	–
Grade 3 (GCS ≤ 8)	30	26	–	–	4	–
**Initial unconsciousness** (yes/no/n. a.)	33/5/4	26/4/4	–	–	5/0/0	–
**Initial pupillary defect** (yes/no/n. a.)	10/31/1	9/25	–	–	0/5/0	–

### Classification Results—MRI Model

We compared six different MRI based models to predict DAI in patients with TBI representing the different ROIs of the atlas for predilection areas. The most significant differences in radiomics features for DAI between patients and controls using DWI were found in the ROIs of corpus callosum, thalamus and basal ganglia.

**Fig. 3:**
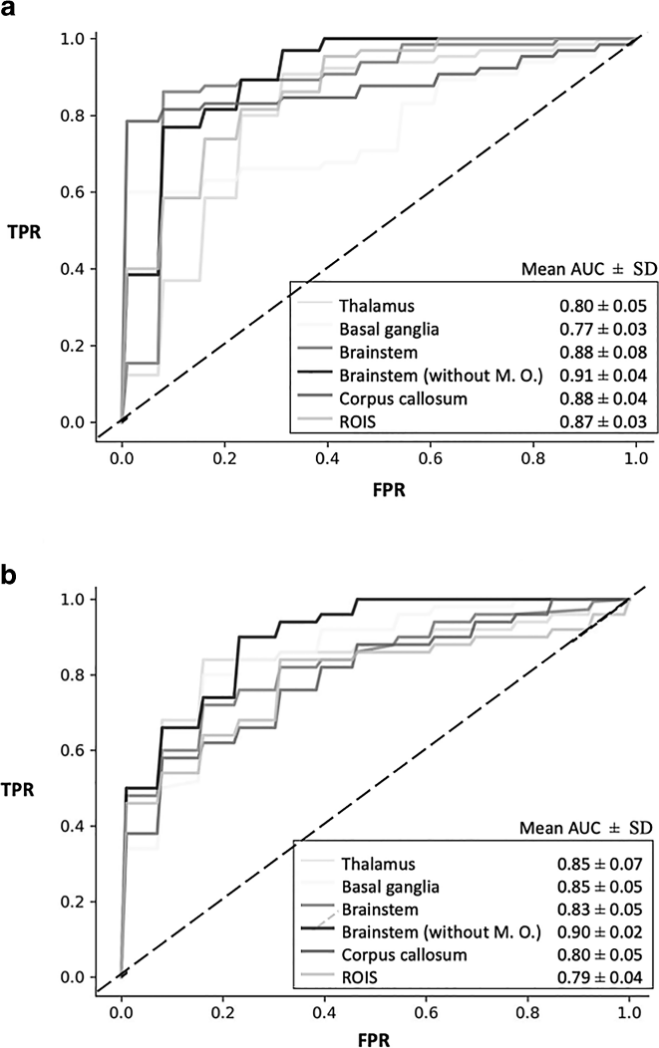
**a** Classification results of the MRI-based models in the training dataset. The best results were achieved using a random forest model. The classifier yielded an area under the receiver operating characteristic curve (AUC) of 0.77 to 0.91 for the different regions of interest. **b** Classification results of the CT-based models in the training dataset. The best performance was achieved for the ROIs of brain stem (AUC 0.9), thalamus (AUC 0.85) and basal ganglia (AUC 0.85)

The best classification results were achieved using a random forest classifier yielding mean receiver operating characteristic curves (AUC) of 0.92 for the corpus callosum using 5 features (entropy, kurtosis, maximum, gray-level co-occurrence matrix (GLCM) autocorrelation, GLCM inverse difference moment normalized), 0.81 for the thalamus using 3 features (kurtosis, median, GLCM cluster shade) and 0.77 for the basal ganglia using 2 features (GLCM inverse difference moment normalized, 90th percentile) in the training set (see Fig. 3a). The different classifiers achieved an accuracy of 80–90% (see Table 2).

**Table 2:** Results of the visualimage classification for MRI and CT by two experienced radiologists and the best performing radiomics classifiers of one anatomic region for each modality and dataset

	Radiologist 1		Radiologist 2		Radiomics Classifier		
	Correct	Incorrect	Correct	Incorrect		Correct	Incorrect
***MRI analysis***							
**Training** **+** **Validation set**					**Validation set** *(Corpus callosum classifier)*		
– Patients with DAI	41	1	41	1		13	0
– Controls	44	0	44	0		12	1
– Total	85	1	85	0		25	1
Accuracy (%)	98	N/A	98	N/A		96	N/A
Concordance (k)	1.0	N/A	1.0	N/A		–	N/A
***CT analysis***							
**Training** **+** **Validation set**					**Validation set** *(Thalamus classifier)*		
– Patients with DAI	19	14	20	13		10	0
– Controls	42	0	42	0		10	3
– Total	61	14	62	13		20	3
Accuracy (%)	81	N/A	83	N/A		87	N/A
Concordance (k)	0.55	N/A	0.55	N/A		–	N/A
**Test set**					**Test set** *(Corpus* *callosum* *classifier)*		
– Patients with DAI	4	1	4	1		3	2
– Controls	3	2	4	1		4	1
– Total	7	3	8	2		7	3
Accuracy (%)	70	N/A	80	N/A		70	N/A
Concordance (k)	0.8	N/A	0.8	N/A		–	N/A

*N/A* not applicable

### Classification Results—CT Model

The most significant CT radiomics features between patients and controls were found in the ROIs of brain stem, thalamus, and basal ganglia. In the training set, the random forest model yielded a mean AUC of 0.90, 0.85 and 0.85 with an accuracy of 84%, 88% and 83% for the prediction of DAI in these areas, respectively (see Fig. 3b and Table 2). In the blinded external test data, the classifier achieved the best result for the ROI of corpus callosum with an AUC of 0.68 (accuracy 70%, sensitivity 60%, specificity 80%) (see Tables 3 and 4). The other ROIs showed lower classification performance (thalamus: AUC 0.52, ACC 40%; brain stem AUC 0.48, ACC 40%; basal ganglia AUC 0.6, ACC 60%) (see Tables 3 and 4). A combination of different anatomical regions and classifiers did not further improve the overall predictive performance.

**Table 3:** Results of the MRI radiomics classifier in the training dataset

TRAINING SET (COLOGNE)					
ROI	Sen [%]*	Spec [%]*	Youden’s index	Acc [%]*	AUC*
Thalamus	83 ± 6	78 ± 6	0.61 ± 0.12	81 ± 4	0.80 ± 0.05
Basal ganglia	60 ± 6	100 ± 0	0.60 ± 0.06	80 ± 3	0.77 ± 0.03
Brainstem	89 ± 15	89 ± 7	0.78 ± 0.16	89 ± 11	0.88 ± 0.08
Brainstem (without M.O.)	92 ± 11	83 ± 11	0.75 ± 0.19	88 ± 6	0.91 ± 0.04
Corpus_callosum	83 ± 8	97 ± 7	0.80 ± 0.11	90 ± 5	0.88 ± 0.04
ROIs	85 ± 11	78 ± 6	0.63 ± 0.17	82 ± 5	0.87 ± 0.03

*Sen* Sensitivity; *Spec* Specificity; *Acc* Accuracy; *AUC* Area under the receiver operating characteristics curve

*Mean of 5‑fold CV ± SD

**Table 4:** Results of the CT radiomics classifier in the training and test dataset

ROI	Sen [%]*	Spec [%]*	Youden’s index	Acc [%]*	AUC*
*TRAINING SET (COLOGNE)*					
Thalamus	84 ± 11	91 ± 6	0.75 ± 0.17	88 ± 6	0.85 ± 0.07
Basal ganglia	82 ± 4	85 ± 5	0.67 ± 0.09	83 ± 2	0.85 ± 0.05
Brainstem	76 ± 9	86 ± 11	0.62 ± 0.20	82 ± 8	0.83 ± 0.05
Brainstem (without M.O.)	88 ± 11	82 ± 7	0.70 ± 0.18	84 ± 2	0.90 ± 0.02
Corpus_callosum	74 ± 11	80 ± 14	0.54 ± 0.25	77 ± 7	0.80 ± 0.05
ROIs	78 ± 13	78 ± 14	0.56 ± 0.17	78 ± 5	0.79 ± 0.04
*TEST SET (FULDA)*					
Thalamus	20	60	–	40	0.52
Basal ganglia	100	20	0.2	60	0.6
Brainstem	20	60	–	40	0.32
Brainstem (without M.O.)	0	80	–	40	0.48
Corpus_callosum	60	80	0.4	70	0.68
ROIs	100	0	0	50	0.3

*Sen* Sensitivity; *Spec* Specificity; *Acc* Accuracy; *AUC* Area under the receiver operating characteristics curve

*Mean of 5‑fold CV ± SD

### Classification Results—conventional Radiological Diagnosis

By visual radiological classification, 41 of 42 patients and all controls were classified correctly In the MRI training set with a high diagnostic accuracy of 98% and high interrater reliability (Cohens kappa, 1.0). In contrast, CT-based analysis by the two raters achieved a lower overall accuracy of 81 and 83% (Cohens kappa, 0.55), comparable to the results of the radiomics classifier. All controls were correctly classified. Nevertheless, 14 of 33 (42%) and 13 of 33 (39%) of DAI patients were classified false negative, illustrating the diagnostic challenges of visual CT analysis (see Tables 3 and 4). In the external test set a diagnostic accuracy of 70, respective 80% was achieved by the two radiologists with a good interrater reliability (Cohens kappa, 0.8) (see Tables 3 and 4).

## Discussion

In the present study, we showed that machine learning models based on radiomics features from MRI and CT can detect structural differences in predilection areas of patients with DAI compared to healthy controls. The developed radiomics classifier achieved a diagnostic performance comparable to conventional image diagnosis by two board-certified radiologists for MRI and CT classification.

As CT usually only shows mild or no specific lesions for DAI, we at first performed a proof-of-concept study to evaluate the general potential of radiomics to assess DAI using MRI, which is the current gold standard for diagnosis and is known to display structural changes on DWI imaging. We then transferred the concept to evaluate the potential of radiomics using CT imaging, as especially a CT based classifier would be of additional value in a routine clinical setting.

For development of the MRI radiomics model, DWI as standard sequence for DAI diagnosis was used. DWI depicts the diffusion along white matter tracts, which can be interrupted due to shearing injuries, and is therefore known to be more sensitive for DAI lesion detection compared to structural MRI, especially in cases with non-hemorrhagic lesions [13]. Besides DWI, MRI sequences with high sensitivity for detection of microbleeds as, e.g., SWI is commonly used. As in our study the MRI radiomics model was used for the proof-of concept with a focus on the development of a CT model, we did not include further MRI sequences. The extension of the MRI model with, e.g., SWI or Diffusion-Tensor-Imaging (DTI) as a special type of DWI, might further improve the model’s performance.

Up to now, in the literature no large studies of radiomics analysis in patients with severe TBI or DAI have been conducted and external testing of the developed models is lacking. In a study by Danilov et al., radiomics analysis was performed on DKI of 31 patients with DAI and statistical significant differences in radiomics features of patients compared to controls were detected. Radiomics analyses showed improved diagnostic results compared to simple average DKI map values, supporting the high sensitivity of radiomics for detection of microstructural changes [21]. Besides the application of radiomics for detection of DAI, the performance was assessed in patients with mild TBI. MRI based texture analysis, was feasible to detect significant structural differences between the hemispheres in patients with mild TBI in comparison to healthy controls [19, 20]. Furthermore, using structural MRI and DTI in a series of 91 athletes with a history of mild TBI and post-concussion syndrome, with radiomics analysis the detection of textural differences between patients and controls was possible with a high sensitivity of 0.8, even though the conventional image analysis did not reveal any prominent findings, highlighting the ability of radiomics to detect additional information. Especially the white matter radiomics features indicating shearing injuries were strongly associated with TBI [33]. These findings are in line with the results from our study, although previous studies used DTI and DKI as special types of diffusion-weighted imaging, which might impede the comparison of the study results.

The most significant differences and best classification results were achieved in the typical white matter predilection areas of DAI as e.g. corpus callosum and brain stem. In comparison, the results of the ROIs at the grey matter/white matter interfaces showed lower performance. As shearing injuries might occur in all areas of the brain the development of a whole brain radiomics model and feature extraction without preselection might further improve our promising results.

In TBI patients, CT-based machine learning models were used to predict the intracranial pressure [34], for automated segmentation and volume assessment of intracranial hematomas [35–39] and midline shift [36]. Furthermore, using clinical and CT imaging data and machine learning models, outcome prediction in patients with TBI was performed with high diagnostic accuracy of up to 91% [40, 41]. Radiomics analyses were also used to predict the enlargement of an intracranial hematoma in TBI over time. In a study of Zhang et al., a CT-based radiomics model combined with clinical features achieved a good performance with an AUC of 0.90 [42]. In a comparable study of Wei et al., a combined clinical radiomics model achieved a high accuracy for hematoma progression prediction in a test dataset with an AUC of 0.88 [43]. These promising results indicate that CT radiomics seems to be generally feasible in TBI patients with good results. To the best of our knowledge, our study is the first to assess the use of CT radiomics in DAI patients.

In our study, the results of the CT classifier were compared to the reading of two board-certified radiologists and furthermore tested in an external independent dataset. The blinded analysis of the test dataset revealed a lower diagnostic accuracy of 40–70% compared to the training dataset (accuracy, 77–88%). Nevertheless, the radiomics models’ performance in the ROI for corpus callosum was comparable to the results of the conventional radiological CT assessment with an overall accuracy of 75%. The other ROIs did show lower accuracy and generally lower sensitivity for DAI diagnosis. One explanation for the lower accuracy in the test data might be the small size of the dataset due to the low availability of cases with DAI patients with eligible full datasets including high quality MRI and CT scans. Furthermore, the independent data set was acquired using different scanners and imaging parameters which might have impaired the performance. As the model indeed showed comparable results to the reading of highly trained specialized radiologists but no superior results, it must be considered that the very subtle changes in the imaging of DAI patients which complicate the visual analysis might also affect and limit the performance of the radiomics classifier. Therefore, the results of the developed CT classifier should be interpretated with caution and need to be evaluated in a larger test cohort to further assess the validity of CT radiomics for DAI diagnosis.

The radiomics classifier achieved comparable results to the experienced readers, but did not show superior performance. This might be due to the very subtle changes in imaging of DAI patients which might also affect the radiomics classifiers performance. We also highly agree that the small dataset might have impaired the classifiers performance.

As up to 20% of CT studies in TBI patients are negative without structural abnormalities [10, 44], conventional radiological image assessment tends to underdiagnose DAI, as was also shown in our study with up to 40% of DAI patients classified as false negative. The developed CT based radiomics model using routinely acquired imaging with good diagnostic performance equivalent to the performance of the radiologists might be used as potential screening tool and AI-based decision support for visual image assessment in TBI patients without the need for further invasive and additional diagnostic procedures. Previous studies showed that AI-based decision support might be especially valuable for radiologists/clinicians in training with less experience to support diagnosis and clinical decision making in challenging cases [45]. Further studies to assess the value of the CT radiomics model as diagnostic support system, preferably comparing the performance of different raters at different levels of expertise with and without AI support are needed. In addition, a CT based radiomics model or decision support system might save resources and provide additional information when no MRI is available or feasible in severely traumatized ICU patient and especially in low- and middle-income countries with a very high incidence of TBI [2, 46].

Besides the high incidence of DAI in cases with severe TBI, structural changes in MRI of patients classified as mild TBI with a higher risk to develop neuropsychological deficits may also be observed in up to 20% of cases [44]. As these patients are usually classified as minor trauma with unremarkable imaging, the treatment of neurocognitive deficits might be delayed. Screening tools and prognostic models for outcome prediction in these cases are rare. CT radiomics analysis might identify patients at risk for the development of further deficits which might lead to earlier diagnosis and treatment. Further studies to evaluate CT radiomics in patients with mild TBI are needed, preferably with a correlation with clinical neuropsychological findings.

In patients with DAI shearing of the white matter tracts leads to axonal degeneration and microscopic changes. Longitudinal MRI studies on DTI of the corpus callosum in DAI patients were able to detect that imaging parameters show changes over time, even up to 12 months after the injury [47]. As structural damage of the brain develops and aggravates over time [7, 8] a radiomics model based on the initial CT scan in a short while after the trauma might still be wrong negative. Nevertheless, as patients tend to present with immediate neurological deficits, the instant impact of the trauma might lead to early changes detected by sensitive radiomics analysis. One option to encounter that problem and gain better insight in imaging parameters in DAI patients, might be the analysis of multiple CT scans over time to detect potential longitudinal changes in the radiomics features.

One limitation of our study is the rather small test dataset and limited number of patients included in the training process. High-quality MRI including SWI and DWI as gold standard for diagnosis of DAI is not always feasible in severely traumatized ICU patients. Therefore, the available training and test dataset comprising patients with eligible CT and MRI imaging for our study are rather small and the results, especially in the test dataset, should be interpreted with caution and further validated in a larger patient cohort.

Another limitation impeding the comparability of the patient and control group is an observed imbalance regarding sex in the two cohorts. This imbalance might have occurred due to the higher incidence of TBI in male patients and due to the limited availability of eligible patients with complete imaging datasets.

As TBI occurs in young patients, e.g., after accidents but is also increasing in the elderly population we included a wide age range in the study to represent this versatile patient cohort. Nevertheless, this may also impair the interpretability of our results.

Another challenge in the interpretation of the results of radiomics analysis is the correlation of the detected imaging features with pathological results and clinical biomarkers. The most important radiomics features detected in our study such as the inverse difference moment of the GLCM and GLCM cluster shade are measures of local homogeneity of an image. A significant difference of these features, representing a difference in the local expression and coherence of voxel intensities between the patient and control group might be an indicator for subtle structural changes due to DAI lesions that are not accessible by visual image analysis. As radiomics analyses has the potential to capture these differences and to provide additional information not accessible by conventional image analysis, radiomics features might help to gain a better insight in pathology of DAI and might be used for individualization of trauma therapy in the future. To enable a possible translation in clinical practice, a better understanding of the biological meaning of these features is crucial. Therefore, further prospective studies correlating imaging features with clinical findings and neurological outcome, preferably in a large dataset from different scanners and institutions are needed.

## Conclusion

MRI and CT based radiomics analysis detects structural differences of radiomics features in patients with DAI compared to controls and offers the potential to support conventional image analysis to improve the diagnosis of DAI. Especially a radiomics based CT classifier might be used as a screening tool for patients with severe and mild TBI. To enable a translation in clinical practice and further assess the clinical value, larger prospective studies with multimodal imaging preferably from different hospitals are needed.

## Data Availability

The datasets used during the current study are available from the corresponding author on reasonable request
